# Transcriptome reveals the gene expression patterns of sulforaphane metabolism in broccoli florets

**DOI:** 10.1371/journal.pone.0213902

**Published:** 2019-03-25

**Authors:** Zhansheng Li, Yumei Liu, Lingyun Li, Zhiyuan Fang, Limei Yang, Mu Zhuang, Yangyong Zhang, Honghao Lv

**Affiliations:** 1 Chinese Academy of Agricultural Sciences, Institute of Vegetables and Flowers, Beijing, China; 2 The Key Laboratory of Biology and Genetic Improvement of Horticultural Crops, Ministry of Agriculture, P. R. China, Beijing, China; Chungnam National University, REPUBLIC OF KOREA

## Abstract

Sulforaphane is a new and effective anti-cancer component that is abundant in broccoli. In the past few years, the patterns of variability in glucosinolate content and its regulation in *A*. *thaliana* have been described in detail. However, the diversity of glucosinolate and sulforaphane contents in different organs during vegetative and reproductive stages has not been clearly explained. In this paper, we firstly investigated the transcriptome profiles of the developing buds and leaves at bolting stage of broccoli (B52) to further assess the gene expression patterns involved in sulforaphane synthesis. The *CYP79F1* gene, as well as nine other genes related to glucorahpanin biosynthesis, *MAM1*, *MAM3*, *St5b-2*, *FMO GS-OX1*, *MY*, *AOP2*, *AOP3*, *ESP* and *ESM1* were selected by digital gene expression analysis and were validated by quantitative real-time PCR (qRT-PCR). Meanwhile, the compositions of glucosinolates and sulforaphane were detected for correlation analysis with related genes. Finally the RNA sequencing libraries generated 147 957 344 clean reads, and 8 539 unigene assemblies were produced. In digital result, only *CYP79F1*, in the glucoraphanin pathway, was up-regulated in young buds but absent from the other organs, which was consistent with the highest level of sulforaphane content being in this organ compared to mature buds, buds one day before flowering, flowers and leaves. The sequencing results also presented that auxin and cytokinin might affect glucoraphanin accumulation. The study revealed that up-regulated expression of *CYP79F1* plays a fundamental and direct role in sulforaphane production in inflorescences. Two genes of *MAM1* and *St5b-2* could up-regulated glucoraphanin generation. Synergistic expression of *MAM1*, *MAM3*, *St5b-2*, *FMO GS-OX1*, *MY*, *ESP* and *ESM1* was found in sulforaphane metabolism. This study will be beneficial for understanding the diversity of sulforaphane in broccoli organs.

## Introduction

In recent years, sulforaphane has attracted much interest due to its anti-cancer activity, and a growing body of epidemiological evidence has shown that increased consumption of sulforaphane or cruciferous vegetables rich in sulforaphane can lower the risk of lung [1], colon [2], pancreatic [3], breast [4], bladder [5] and prostate [6] cancers as well as some geriatric diseases such as Alzheimer's disease [7] and cardiovascular disease [8, 9]. The chemoprotective function of sulforaphane is due to its ability to induce phase II detoxification enzymes [10, 11], directly resulting in cancer cell apoptosis [12, 13].

Sulforaphane is an isothiocyanate, and it can be synthesized from glucoraphanin through hydrolysis by myrosinase when broccoli is chewed, mechanically damaged, digested by humans, or bitten by insects [14, 15]. Glucoraphanin (4-Methylsulfonylbutyl glucosinolate) is a glucosinolate mostly found in *Brassica* vegetables, such as broccoli, cabbage (green and red), Chinese kale, Brussels sprouts, kohlrabi, collards, and turnip [16–18]. Among the crucifers tested, broccoli has been reported to be rich in glucoraphanin, and the regulation of glucosinolate synthesis has been largely reported in *A*. *thaliana* [19–22].

Glucosinolates are mainly synthesized from amino acids Met, Phe and Trp, which accordingly give rise to three groups of glucosinolates: aliphatic glucosinolates, benzenic glucosinolates and indolic glucosinolates [15, 22–24]. Regulation genesof glucosinolate and the pathway have been successfully identified in *Arabidopsis* [23, 25–27].Glucoraphanin belongs to aliphatic glucosinolate derived from Met. In the process of chain elongation, it starts with deamination by a BCAT4 giving rise to a 2-oxo-4-methylthiobutanoic acid. The 2-oxo-4-methylthiobutanoic acid then enters a cycle of three successive transformations: condensation with acetyl-CoA by *MAM1* and *MAM3*, isomerization by *IPMI-SSU2*, *3*, and oxidative decarboxylation by *IPM-DH*, generating 2-Oxo-6-methylthiohexanoic acid [16, 28].

A total of 13 enzymes, representing five different biochemical steps in the formation of the glucosinolate core structure, have been characterized [24, 29]. For the core biosynthetic pathway of aliphatic glucosinolates, *CYP79F1* (Met1-6), *CYP79F2* (Met 5, 6), *CYP83A1*, *GSTF11*, *GSTU20*, *GGP1*, *SUR1* (*C-S lyase*), *UGT74C1*, *SOT17* (*AtSTb*), and *SOT18* (*AtSTb*) play distinct roles in oxidation, conjugation, C-S cleavage, glucosylation and sulfation functions, then 2-oxo-6-methylthiohexanoic acid and dihomomethionine are transferred to 4-methylthiobutyl glucosinolate (glucoerucin). Finally, glucoerucin is oxidized and changed into glucoraphanin by *FMO-GSOX1-5* [26, 30].

The following process is secondary modification, and the biological activity of glucosinolates is determined by the structure of the side chain [22, 27]. In aliphatic glucosinolates, 4-methylthiobutyl actually is the precursor of glucoraphanin, is catalyzed to generate 3-pentenyl glucosinolate (gluconapin) by *GS-ALK*, as well as 4-benzoyloxybutyl glucosinolate by *GS-OHB*. Glucoraphanin can also be hydrolyzed to sulforaphane catalyzed by myrosinase (*MY*) or, depending on pH, more sulforaphane is generated in an alkaline environment [28, 31, 32]Together with side-chain elongation, secondary modifications are responsible for more than 132 known glucosinolate structures [33, 34], of which there have been 56 putative genes identified in glucosinolate pathway of *B*. *oleracea* (http://www.ocri-genomics.org/cgi-bin/bolbase/pathway_detail.cgi?entry=map00966) [35], and 110 in *B*. *rap*a (http://brassicadb.org/brad/glucoGene.php) [36, 37].

By 2010, approximately 29 genes have been found in aliphatic glucosinolate pathway [26, 38–40]. Glucosinolate synthesis and its regulation mechanism has been revealed mostly in *Arabidopsis*. However, glucosinolates are affected by many factors, such as genotypes, organs, development stages, cultivation conditions, soil microbes, and environments [22, 27, 31]. Some research and our previous work have reported that the significant differences of sulforaphane and glucoraphanin happened in different organs of *Brassica* vegetables [16, 28, 41]. But there are few reports that can explain the diversity of sulforaphane contents in different broccoli organs at various developmental stages [42, 43]. And one of the best methods to elucidate these mechanisms is to study them at the molecular level by transcriptome analysis.

In our study, it was found that the contents of glucoraphanin and sulforaphane were both in a high level with significant differences in developing buds. So the buds at bolting stage were chosen and carried out by transcriptome analysis for exploring the gene expression patterns of sulforaphane. The aims of our research were to (i) identify and validate differential expression of specific genes in developing buds (LN_B1-B4) and leaves (LN_F) individually, and (ii) find genes related to sulforaphane metabolism. Our results would provide new insights into explanation of sulforaphane accumulation in different organs of broccoli.

## Materials and methods

### Plant material

**Fig 1:**
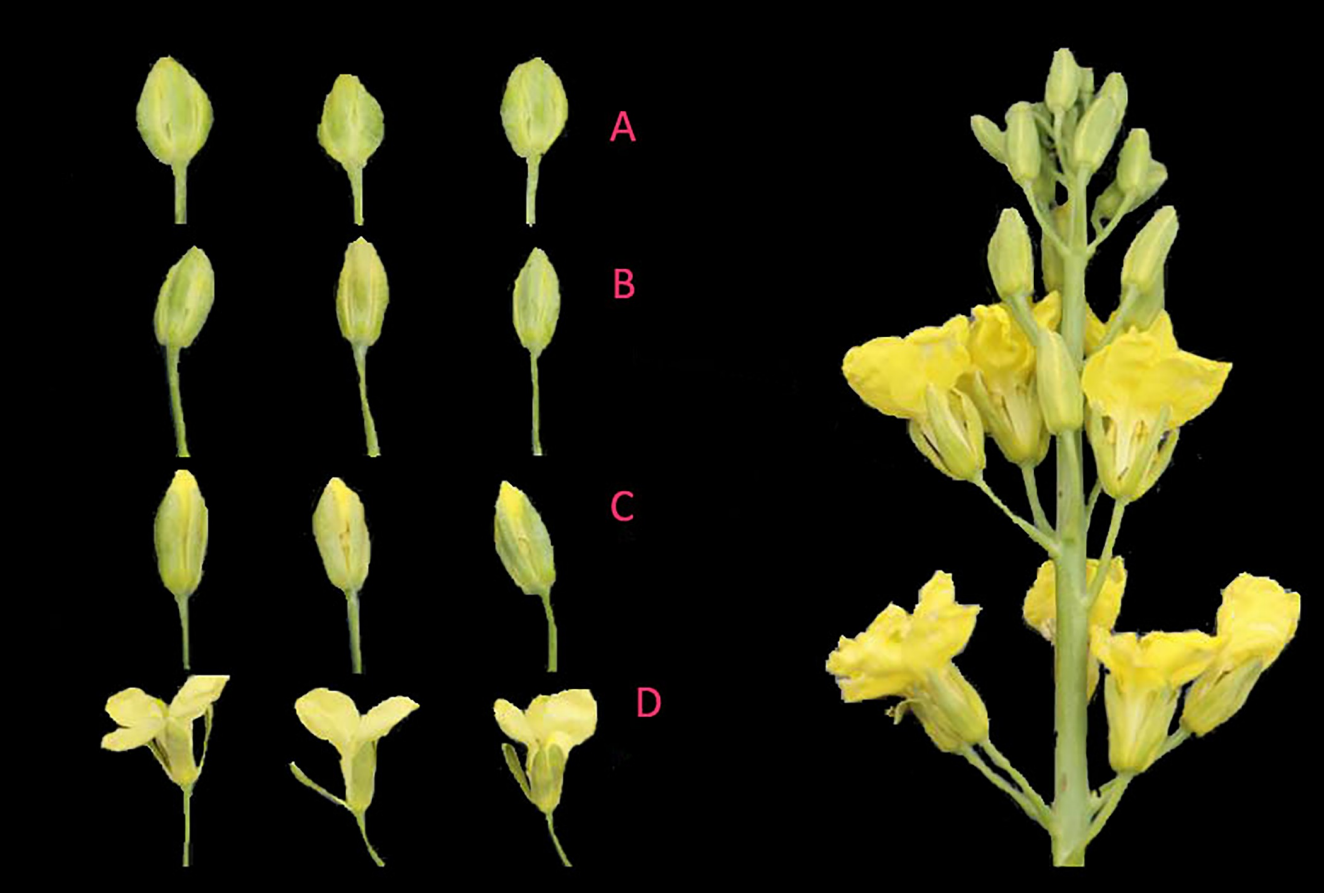
The developmental buds of young buds (A), mature buds (B), buds one day before flowering (C) and flowers (D) at bolting stage.

Broccoli inbred line B52 was cultured and treated using the method described in our previous research, and this inbred line was bred at the Institute of Vegetables and Flowers, Chinese Academy of Agricultural Sciences (CAAS-IVF) [41]. All plants were planted in greenhouse on August 2, 2015, florets formed on October 15 and bolting on November 22. At the same time, the developmental buds and leaves (LN_F) were collected at bolting stage, and the organs were young buds (LN_B1), mature buds (LN_B2), buds one day before flowering (LN_B3) and flowers (LN_B4) (Fig 1).

### Library construction, sequencing and bioinformatics analysis

Total RNA was extracted from each sample by using TRIzol reagent (Invitrogen, CA, USA), and its quality was monitored on 1% agarose gels and assessed by a Bioanalyzer 2100 system (Agilent Technologies, Santa Clara, CA, USA) with a minimum RNA integrity number (RIN) of 7.0. Sequencing libraries were generated using the NEBNext Ultra RNA Library Prep Kit for Illumina (NEB, USA), and index codes were assigned to each sample. Library quality was assessed on the Agilent Bioanalyzer 2100 system. Clustering of the index-coded samples was performed on a cBot Cluster Generation System using the TruSeq PE Cluster Kit v3-cBot-HS (Illumina). After cluster generation, the prepared libraries were sequenced on an Illumina HiSeq 2500/4000 platform (Illumina, Inc., San Diego, CA, USA), which was conducted by Beijing Allwegene Technology Co., Ltd, China. Before assembly, raw reads of the cDNA libraries were filtered to remove adaptor sequences, low-quality reads containing poly-N and sequences with more than 5% unknown nucleotides. After transcriptome assembly, each unigene was annotated using five databases [44, 45]: NCBI non-redundant protein (Nr), Eukaryotic Ortholog Groups (KOG), Protein family (Pfam), Swiss-Prot, and Kyoto Encyclopedia of Genes and Genomes (KEGG) databases. Blast all software was used to predict and classify the KOG and KEGG pathway-associated unigenes [46, 47], employing BlastX (v.2.2.28C) with an E-value of less than 1e^-5^. Gene Ontology (GO) annotations were analyzed using GOseq [48].

### Analysis of differentially expressed genes

All quenching reads for five samples were remapped to the reference sequences using RSEM software, and the abundance of each assembled transcript was evaluated using FPKM [49–50]. For genes with more than one alternative transcript, the longest transcript was selected to calculate the FPKM. The DESeq package (ver.2.1.0) was employed to detect DEGs between sample pairs (LN_B1 versus LN_F, LN_B2 versus LN_F, LN_B3 versus LN_F and LN_B4 versus LN_F [51, 52]. The false discovery rate (FDR) was applied to correct the *p*-value threshold in multiple tests [52]. An FDR-adjusted *p*-value (*q*-value) ≤ 0.05 and a |log2 Fold Change| > 1 were used as the thresholds for identifying significant differences in gene expression. For convenience, DEGs with higher expression levels in buds compared to leaves were designated up-regulated, whereas those with lower expression were designated down-regulated [50].

### Candidate glucosinolate genes selection and certification of relative expression

To verify the reliability of the expression analysis, ten candidate glucosinolate genes of *MAM1*, *MAM3*, *CYP79F1*, *St5b-2*, *FMO GS-OX1*, *MY* (*TGG1*), *AOP2* and *AOP3*, *ESP* and *ESM1* were selected and quantified by real-time PCR. The primers for these genes were listed in Table 1. Samples of developing buds and leaves were gathered, and qRT-PCR analysis was performed by the method described in our previous study [41], qRT-PCR was carried out using SYBR Premix Ex TaqII (Tli RNaseH Plus; TAKARA BIO, Inc., Shiga, Japan) on an ABI 7900HT (Applied Biosystems, Carlsbad, CA, USA).

**Table 1:** The qRT-PCR genes related sulforaphane metabolism and their primers.

No	Gene names	Primer sequences
1	*MAM1* Forward primer	GAGTAGACATCATGGAAGTCGGTT
	*MAM1* Reverse primer	AAGTCGCCTCAATGTCTCTATGTT
2	*MAM3* Forward primer	CGAAGTGACGATCAACGGAA
	*MAM3* Reverse primer	GACATTTCAAAGCCATCACGAC
3	*CYP79F1* Forward primer	GTCACGCCAGACGAAATCAAA
	*CYP79F1* Reverse primer	GCACAAGCCTGTCTTTTCCAACT
4	*FMO GS-OX1* Forward primer	GGAAAGCAGATCCATAGCCACA
	*FMO GS-OX1* Reverse primer	CATAGATTGTTTTGGGGCACTG
5	*AOP2* Forward primer	AGTAAGAGTGACCGAGAAAAAGAGG
	*AOP2* Reverse primer	GCGACCAGCTTCTGAGTGATAG
6	*AOP3* Forward primer (homologous domain)	AGGTGAAGACCAAAGAGGGGAA
	*AOP3* Reverse primer (homologous domain)	TCGGTGATACGGTGAAGGGA
7	*MY* Forward primer	GCTGTGAGGTGTGAGCGGTAA
	*MY* Reverse primer	GTCTCATAAGTTAGAATTGACGCCA
8	*St5b-2* Forward primer	CCCATATACCCAACGGGTCG
	*St5b-2* Reverse primer	CCCATGAACTCAGCCAACCT
9	*ESP* Forward primer	GATCAAGGTGGGGCAGAAAG
	*ESP* Reverse primer	AAGGTTTCGCTCCTGTAGTCTCTA
10	*ESM1* Forward primer	AAGATCTTCCACAAACCTATTG
	*ESM1* Reverse primer	TTTGTATTCTTGTCTCACGATC
11	actin-12 Forward primer	GGCTCTATCTTGGCTTCTCTCAGT
	actin-12 Reverse primer	CCAGATTCATCATACTCGGCTTT

### Investigation of glucosinolate genes associated with sulforaphane

To gain overall insight into differential gene expression patterns between developing buds and leaves. Ten regulated genes related to the sulforaphane pathway were chosen for confirmation by quantitative real-time PCR (qRT-PCR). These genes are *MAM1*, *MAM3*, *CYP79F1*, *St5b-2*, *FMO GS-OX1*, *AOP2*, *AOP3*, *MY*, *ESP* and *ESM1*.

### Extraction and determination of sulforaphane and glucoraphanin

Five samples were pretreated and dried in a lyophilizer, HPLC and UHPLC–Triple–TOF–MS methods were used for determination of sulforaphane and glucoraphanin separately. The extraction and determination methods of sulforaphane are thoroughly described in our previous study [41, 53].

The methods for analysis of glucoraphanin and the other glucosinolates was carried out by using UHPLC-Triple-TOF-MS. Samples were extracted using 70% methanol and injected after concentration of the standard glucoraphanin. UPLC BEH C_18_ (2.1 mm × 100 mm, 1.7 μm) column was selected with acetonitrile-water (both 0.1% formic acid) as mobile phase. Chromatographic separation was achieved under gradient elution in 10 min. In ESI negative ion mode, TOF-MS scan-IDA-Product ion scan was performed to acquire both MS and MS/MS information from one injection. Based on high resolution TOF-MS, accurate masses of molecular ions and fragment ions were obtained for high accuracy-identification.

## Results

### Sequencing, assembly and functional annotation

**Fig 2:**
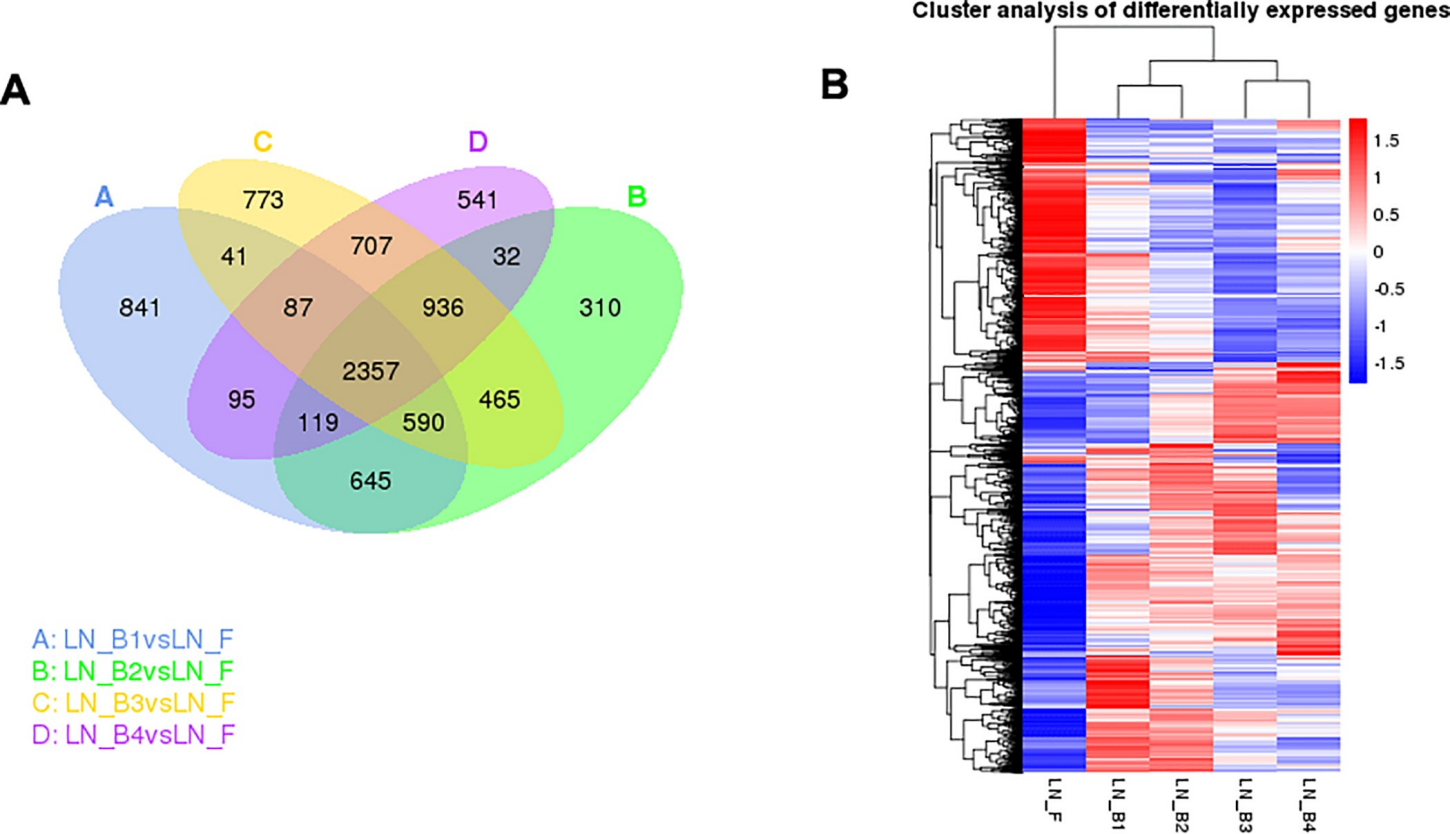
**Venn diagram comparisons (A) and cluster analysis of differentially expressed genes between leaves and developmental buds (B).** Venn diagram comparison of differentially expressed genes between leaves and developmental buds at bolting stage. Hierarchical cluster analysis of differentially expressed genes among genotypes. The color key represents Lg (RPKM + 1). Red indicates high relative expression and blue indicates low relative expression. LN_F denotes leaves and LN_B (1~4) denotes developmental buds of broccoli at bolting stage.

**Fig 3:**
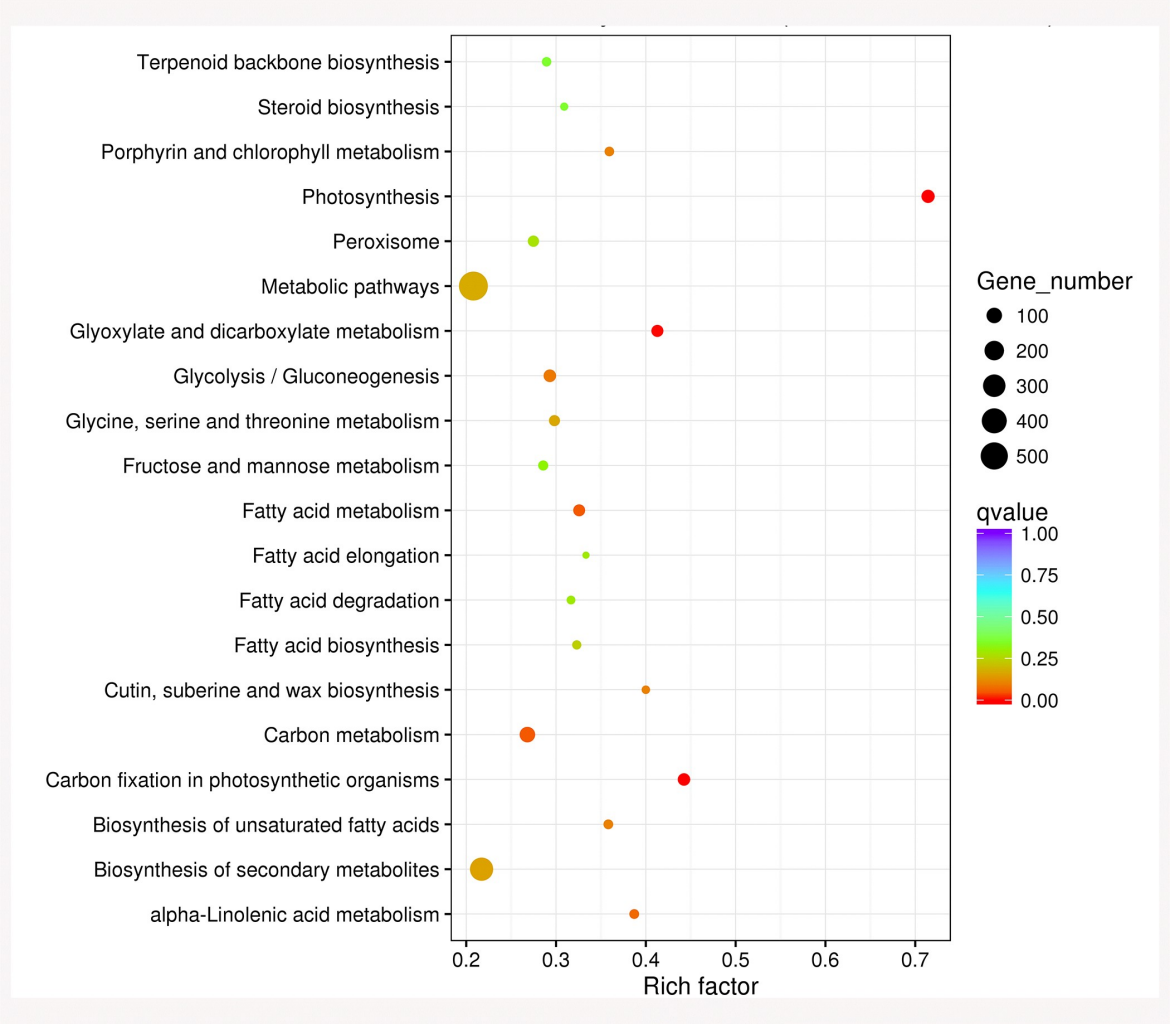
The top 20 KEGG pathways with the highest representation of common DEGs from pairwise comparisons between developmental buds and leaves.

A pooled cDNA library of five samples of developing buds and leaves was analyzed on the Illumina HiSeq 2500/4000 platform (Illumina, Inc., San Diego, CA, USA). The library generated 147.96 million raw reads (Tables 2 and 3), and the assembled raw reads (>95.23%) had Phred-like quality scores at the Q20 level (an error probability of 0.01–0.02%). Finally 48 852 unigenes of 150 bp generated based on PE150. The Gene Ontology (GO) database assigned 27 606 unigenes into 30 functional categories. The largest proportion was represented by biological process (GO 0008150, 11.86%) and metabolic process (GO 0008152, 12.39%; S1 Fig). In total, 6656 unigenes were categorized into 4 Clusters of Orthologous Groups of Proteins (COG) classifications (S2 Fig), which was shown and validated by Venn diagram comparisons (Fig 2A) and cluster analysis of differentially expressed genes between leaves and developmental buds (Fig 2B). The 3450 assembled sequences were mapped to the reference canonical pathway in the Kyoto Encyclopedia of Genes and Genomes (KEGG). In the top 20 KEGG pathways, the pathway most strongly represented by the mapped unigenes was biological process and metabolism (KO 03010, 263 unigenes) (Fig 3).

**Table 2:** Comparison of reads and reference sequence.

Sample	LN_F	LN_B1	LN_B2	LN_B3	LN_B4
**Total reads**	70435770	79567320	51953284	45781558	48176756
**Total mapped**	50627502 (71.88%)	57043101 (71.69%)	37400419 (71.99%)	33219629 (72.56%)	33514062 (69.56%)
**Multiple mapped**	1760525 (2.5%)	1385966 (1.74%)	1048692 (2.02%)	894641 (1.95%)	740476 (1.54%)
**Uniquely mapped**	48866977 (69.38%)	55657135 (69.95%)	36351727 (69.97%)	32324988 (70.61%)	32773586 (68.03%)
**Read-1**	25396488 (36.06%)	28907373 (36.33%)	18828753 (36.24%)	16742405 (36.57%)	17539166 (36.41%)
**Read-2**	23470489 (33.32%)	26749762 (33.62%)	17522974 (33.73%)	15582583 (34.04%)	15234420 (31.62%)
**Reads map to '+'**	24496371 (34.78%)	27873454 (35.03%)	18184082 (35%)	16172019 (35.32%)	16390884 (34.02%)
**Reads map to '-'**	24370606 (34.6%)	27783681 (34.92%)	18167645 (34.97%)	16152969 (35.28%)	16382702 (34.01%)
**Non-splice reads**	32180436 (45.69%)	34794098 (43.73%)	23101224 (44.47%)	21927513 (47.9%)	21693113 (45.03%)
**Splice reads**	16686541 (23.69%)	20863037 (26.22%)	13250503 (25.5%)	10397475 (22.71%)	11080473 (23%)

**Table 3:** Sequencing and assembly statistics for the 10 transcriptomes of the B52 inbred line at bolting stage.

Sample	Raw Reads	Raw Bases	Clean Reads	Clean Bases	Error Rate	Q20	Q30	GC Content
**LN_F_1**	36374237	5.45Gb	35217885	5.28Gb	0.01%	99.26%	97.90%	46.15%
**LN_F_2**	36374237	5.45Gb	35217885	5.28Gb	0.01%	97.27%	94.13%	46.23%
**LN_B1_1**	41132570	6.16Gb	39783660	5.97Gb	0.01%	99.27%	97.94%	45.84%
**LN_B1_2**	41132570	6.16Gb	39783660	5.97Gb	0.01%	97.31%	94.23%	45.90%
**LN_B2_1**	26796422	4.01Gb	25976642	3.9Gb	0.01%	99.26%	97.90%	45.70%
**LN_B2_2**	26796422	4.01Gb	25976642	3.9Gb	0.01%	97.46%	94.51%	45.75%
**LN_B3_1**	23634550	3.54Gb	22890779	3.43Gb	0.01%	99.22%	97.81%	45.09%
**LN_B3_2**	23634550	3.54Gb	22890779	3.43Gb	0.01%	97.45%	94.49%	45.17%
**LN_B4_1**	25283977	3.79Gb	24088378	3.61Gb	0.01%	98.75%	96.66%	45.49%
**LN_B4_2**	25283977	3.79Gb	24088378	3.61Gb	0.02%	95.23%	89.81%	45.75%

### Identification and annotation of differentially expressed genes

Approximately 29.88–51.52 million 150 bp paired-end reads were generated through RNA sequencing (S3 Fig). Transcript levels were calculated using fragments per kilobase per million reads (FPKM; Table 3). The GC content from the 10 libraries ranged from 45.09 to 46.23%, and the Q30 values (reads with an average quality scores > 30) were all in the range of 89.81 to 97.90%, indicating that the quality and accuracy of sequencing data were sufficient for further analysis (Table 2). The percentage of sequenced reads from all libraries that remapped to the assembled reference transcripts was nearly ≥ 70% (Table 1). According to the cabbage reference genome, 8539 genes of 45758 unigenes were functionally annotated with an e-value ≥ 1e^-5^ in at least one database.

**Fig 4:**
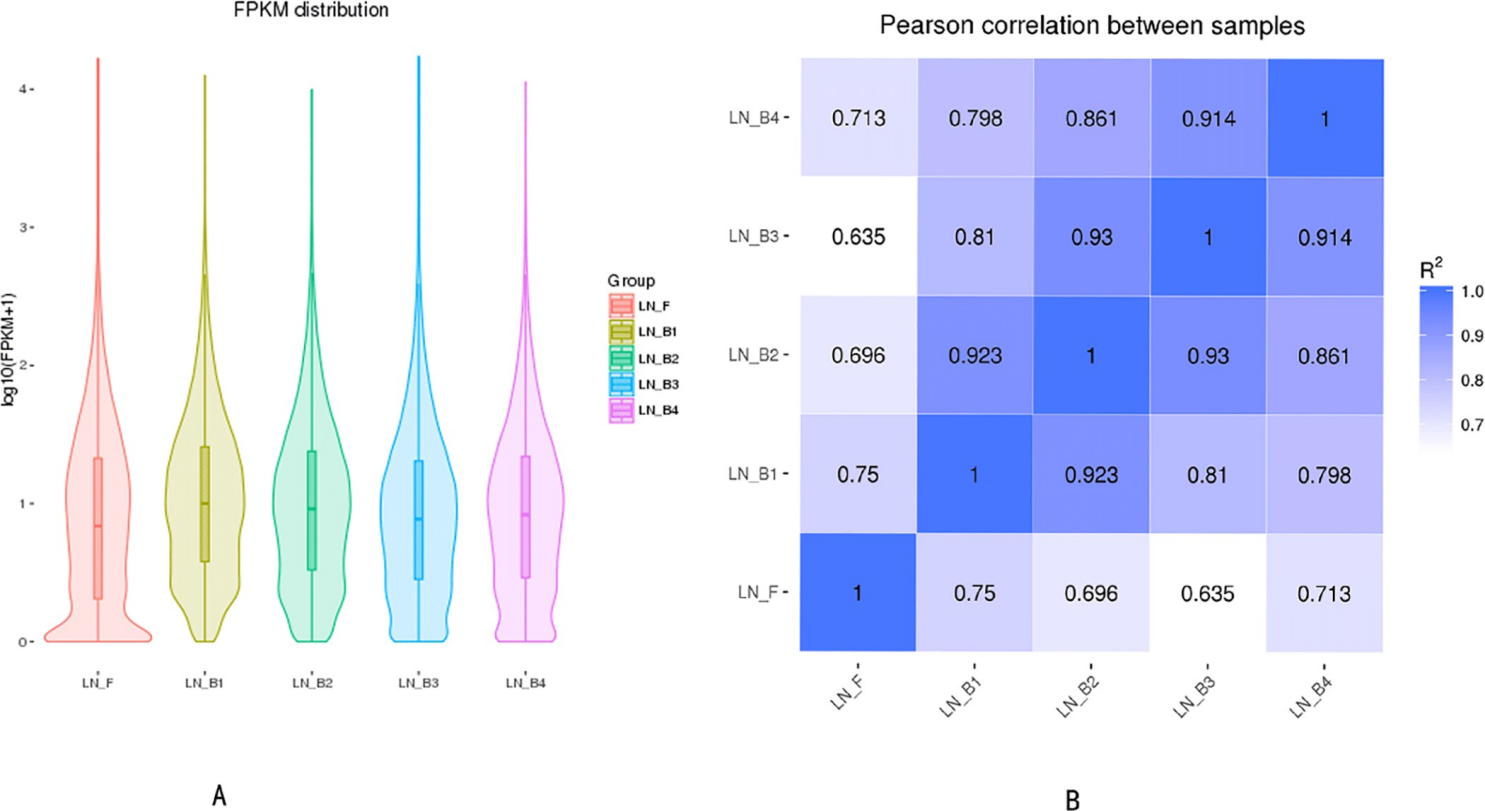
**Violin plot of the normalized FPKM values for gene expression in different groups (A).** Absolute magnitude (log) of the divergence of absolute magnitude of log (FPKM+1) resulting from leaves (LN_F), young buds (LN_B1), mature buds (LN_B2), buds one day before flowering (LN_B3) and flowers (LN_B4) of broccoli at bolting stage. **Pearson correlation between samples of developmental buds (LN_B1**~**4) and leaves (LN_F) (B)**.

Differential expression in young buds (LN_B1), mature buds (LN_B2), buds one day before flowering (LN_B3), flowers (LN_B4) and leaves (LN_F) of broccoli at bolting stage (FPKM > 5.0 in at least one treatment group, fold change ≥2.0, *P* ≤ 0.05) was found for 4775 to 5956 genes. Of these, 2534 to 3101 were up-regulated and 2000 to 2974 were up-regulated in all four groups of developing buds versus leaves (Table 4). The detailed gene numbers at different interval are shown in Table 5, and most genes were within an FPKM Interval 0~1 (49.85%-58.35%), particularly in leaves, followed by the buds one day before flowering, flowers and mature buds (Table 4). As shown in Fig 4A, we found that low expression genes were enriched in leaves, followed by buds one day before flowering, flowers, mature buds and young buds. However, young buds had higher overall gene expression, the second was mature buds, flowers, buds one day before flowering, and leaves showed the least (Fig 2A). Pearson correlations between five organs were calculated to investigate relationship of developing buds and leaves (Fig 4B). There was a gradual decrease in developing buds from young buds to flowers (LN_B1~4), which was consistent with the phenotype. In contrast, leaves displayed a varying relationship, which were most similar to young buds. All the differentially expressed genes were annotated by the databases described above.

**Table 4:** The number of differentially expressed genes between different pairs samples.

Groups/samples	Total number	Up-regulated	Down-regulated
**LN_B1 vs LN_F**	4775	2775	2000
**LN_B2 vs LN_F**	5454	3101	2353
**LN_B3 vs LN_F**	5956	2982	2974
**LN_B4 vs LN_F**	4874	2534	2340

**Table 5:** The statistics of gene numbers at different interval level.

FPKM Interval	LN_F	LN_B1	LN_B2	LN_B3	LN_B4
0~1	35975(58.35%)	30733(49.85%)	32042(51.97%)	33284(53.99%)	33231(53.90%)
1~3	4779(7.75%)	5840(9.47%)	5558(9.02%)	5628(9.13%)	5349(8.68%)
3~15	10372(16.82%)	12451(20.20%)	12218(19.82%)	12405(20.12%)	12054(19.55%)
15~60	7365(11.95%)	9008(14.61%)	8396(13.62%)	7332(11.89%)	7832(12.70%)
>60	3159(5.12%)	3618(5.87%)	3436(5.57%)	3001(4.87%)	3184(5.16%)

### Investigation of the glucosinolate genes associated with sulforaphane metabolism

**Fig 5:**
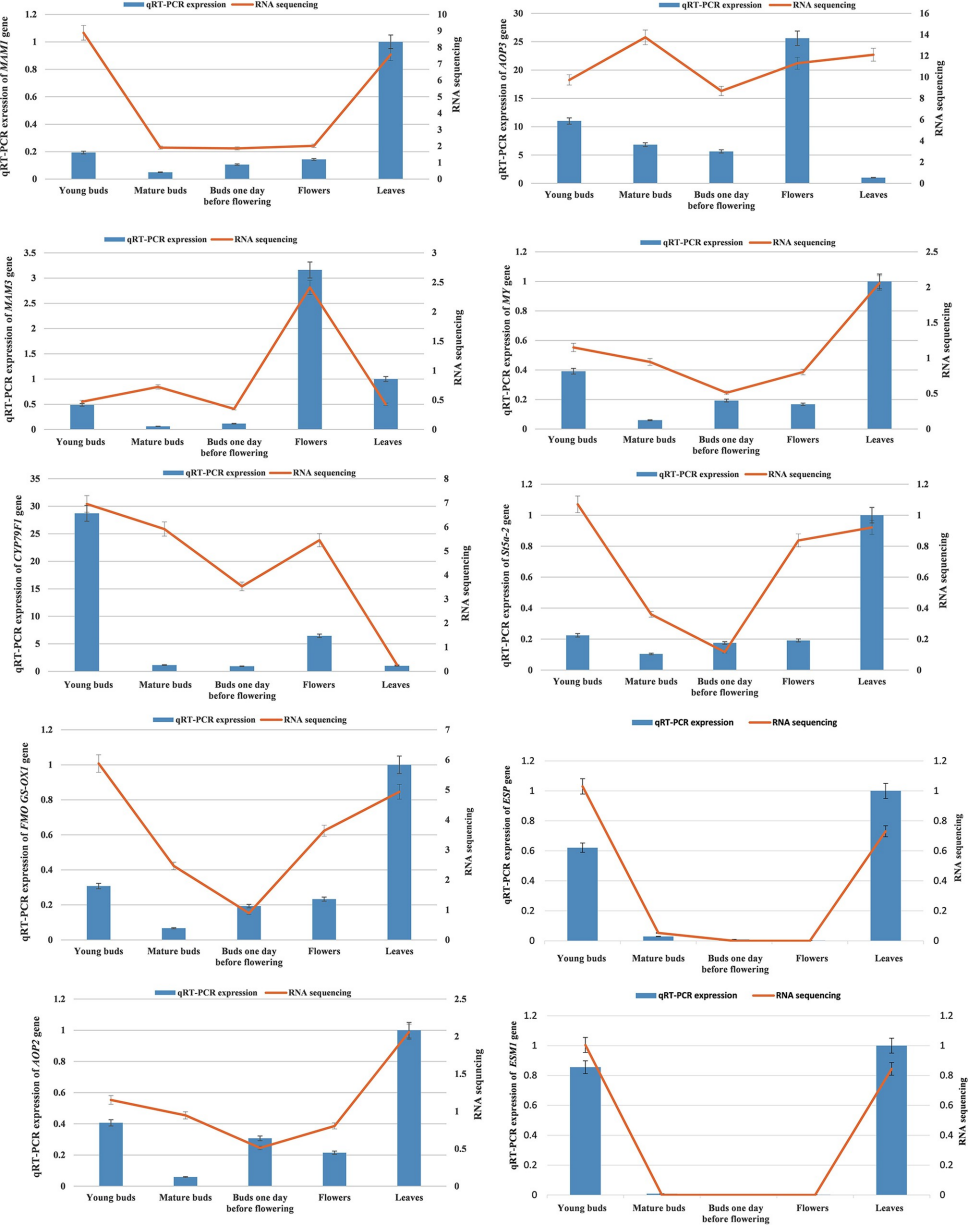
RNA sequencing and qRT-PCR results of the expression genes related with sulforaphane metabolism.

The expression of the glucosinolate genes, including glucosinolate core genes and secondary metabolic genes were confirmed by qRT-PCR. Most of these genes showed similar trends in RNA sequencing and qRT-PCR (Fig 5). In this study, ten genes were investigated and compared with sulforaphane concentrations measured by HPLC. It was found that unlike *CYP79F1* and *AOP3*, the genes *MAM1*, *MAM3*, *St5b-2*, *FMO GS-OX1*, *MY*, *AOP2*, *ESP* and *ESM1* displayed a low expression level compared to the leaf control. There was a significantly higher expression of *CYP79F1* in the young buds compared to the other organs at this stage, following by flowers and mature buds and buds one day before flowering, and leaves had at the lowest expression level (Fig 5). *AOP3* also showed a high gene expression similarly to *CYP79F1*, but the highest-expressing organ was flowers, and second were young buds followed by mature buds and buds one day before flowering, with leaves having the lowest level.

**Fig 6:**
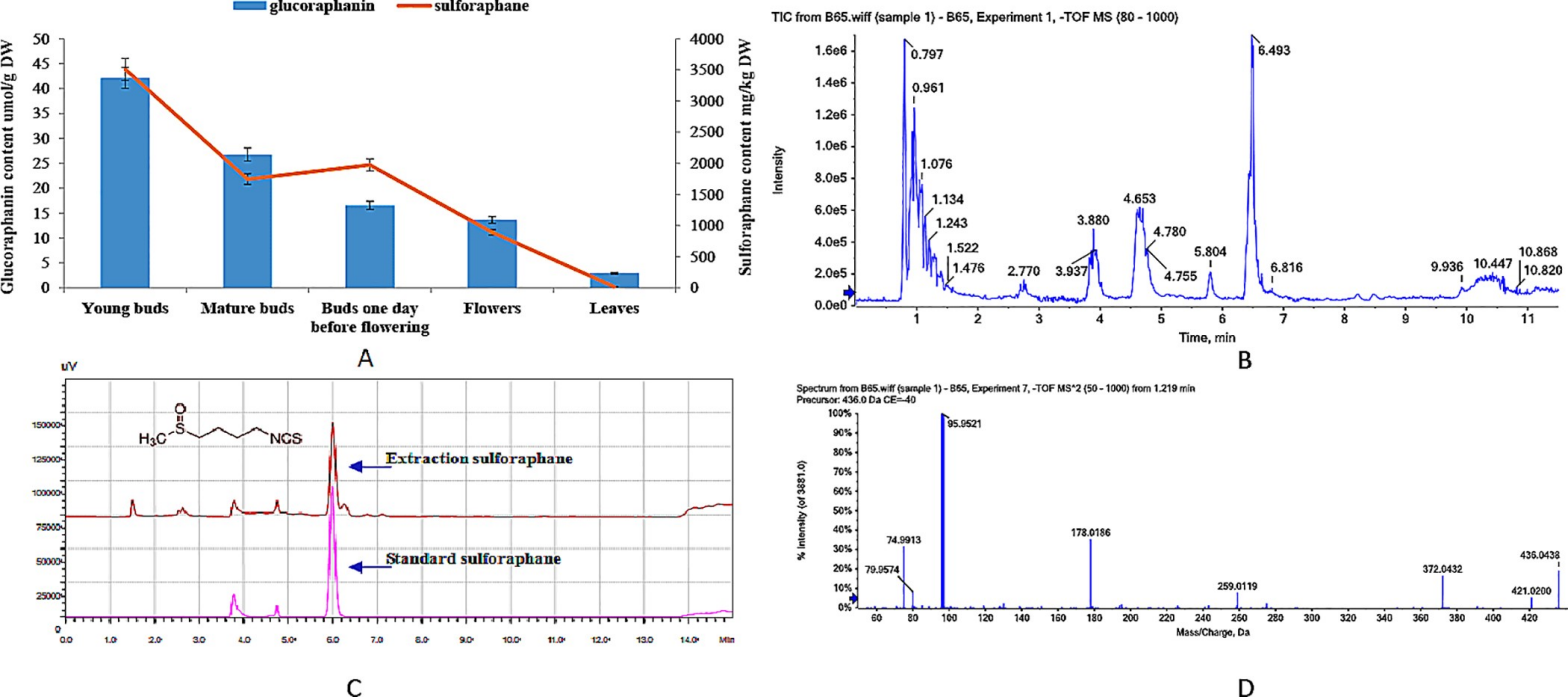
Sulforaphane and glucoraphanin concentrations detected in different organs of broccoli at bolting stage (A). Chromatography of sulforaphane (C) and TIC Chromatograph of glucosinolate (B) corresponding to glucoraphanin spectrum (RT) (D).

The same changing trends of sulforaphane and glucoraphanin happened to B52 at bolting stage, and there was an obvious decrease in sulforaphane and glucoraphanin concentrations from young buds to leaves (Fig 6A). Also there was a sharp decrease to mature buds from young buds, then another decrease from flowers to leaves. The corresponding sulforaphane contents were 3370.44, 2140.34, 1323.98, 1090.46, 235.82 mg/kg DW, respectively (Fig 6A and 6C). The corresponding contents of glucoraphanin were 43.83, 21.82, 24.65, 11.14 and 2.27 μM/g DW (Fig 6A and 6D). So the generation efficiency of sulforaphane from glucoraphanin was 30.3% to 58.6% in these organs. Except the buds one day before flowering, the other organs showed the similar efficiency, suggesting there should be no difference of myrosinase activity (ESM1) in catalyzing glucoraphanin into sulforaphane. At the same time, another 11 glucosinolates were detected in our study (Fig 6B), and gluconapin, glucotropaeolin, progoitrin and sinigrin were not determination. The result provided a good evidence for previous reports. This result showed the pattern of sulforaphane accumulation in different organs was consistent with our previous reports [41, 53].

## Discussion

### The glucosinolate pathway and sulforaphane metabolism

In the past 30 years, 16 natural glucosinolates in broccoli and 26 glucosinolates in *A*. *thaliana* have been elucidated. The total number of documented glucosinolates from plants has been 122 types [54–56].

The aliphatic pathway, encompassing 29 genes in *Arabidopsis*, was reviewed in 2010 [23, 31, 57]. Homologs for most of these genes can be found in broccoli, but different copies and variations are usually found in *Brassica* plants, such as AOP family genes [58, 59], which are responsible for the conversion of glucoraphanin to gluconapin in *Arabidopsis*. There are 3 *AOP* copies in broccoli, of which one is functional and two are mutated, whereas three genes in *B*. *napa* are functional [35]. According to sequence alignments acids, the *AOP1* gene has an extra intron in exon 2, produces a smaller predicted protein and may not be functional [58, 60]. The *AOP2* gene has few base changes and no function, and there is a large deletion in exon 2 in *AOP3*, but this gene might still retain its function. *AOP3* was not found in *B*. *napa* [58, 60, 61]. Another gene, *FMO GS-OX1*, is responsible for the conversion of glucoerucin into glucoraphanin, which is important for sulforaphane generation. However, there are few differences between broccoli plants [39]. In *Arabidopsis*, the MAM family contains three tandemly duplicated and functionally diverse members (*MAM1*, *2*, *3*). *MAM1* and *MAM2* catalyze the condensation of the first two elongation cycles for the synthesis of the dominant C3 and C4 side chain aliphatic glucosinolates, respectively [62, 63], whereas *MAM3* is assumed to contribute to the production of all glucosinolate chain lengths [22]. However, in *B*. *rapa* and *B*. *oleracea*, *MAM1*/*MAM2* genes experienced independent tandem duplication to produce C6 and C5 orthologs, respectively [24, 35]. In addition to the *MAM3* homologs in *Brassica*, at least two *MAM3* genes seem to be involved the C-side chain size: *BoGSL-PRO* and *BoGSL-ELONG*, determining glucosinolate of C3 and C4 side chains, respectively [60, 64].

In this study, the genes of *MAM1*, *MAM3*, *CYP79F1*, *St5b-2*, *FMO GS-OX1*, *MY*, *AOP2*, *AOP3* (homologous domain), *ESP* and *ESM1* were detected and analyzed by qRT-PCR. Combined with the transcriptome data, the study would help us to reveal the gene expression patterns of sulforaphane in the developmental buds at bolting stage.

Glucoraphanin belongs to C4 glucosinolate, which might be produced by *MAM1*/*MAM2* genes. In *B*. *rapa*, *MAM3* plays an important role in accumulation of C5 glucosinolates, such as glucobrassicianapin [35, 37]. However, our results showed that leaves had a higher level of *MAM1* gene expression than the developing buds, which was depending on the cultivar of broccoli (Fig 5), and all the materials in this study had a low level of *MAM1* as well as *MAM3* expression, with the exception of the flowers, which had a slightly higher expression. This was consistent with the transcriptome results, which showed no significant differences between *MAM1* and *MAM3*. In this study, the sulforaphane and glucoraphanin contents in developing buds were inversely correlated with the developmental stages,which might be caused by low *MAM1* gene expression after bolting [31, 65].

*A*. *thaliana* with the *CYP79F2* gene knocked out showed substantially reduced long-chain aliphatic glucosinolates and increased short-chain aliphatic glucosinolates, and *CYP79F1* increases both long- and short-chain aliphatic glucosinolates [26, 29]. In our study, *CYP79F1* (*Bo5g021810*-*4*.*2906*) was only significantly up-regulated in aliphatic glucosinolate biosynthesis, howeverthis up-regulation was not found in the other developing buds and leaves. Therefore, up-regulation of the *CYP79F1* gene might be one of the reasons for causing sulforaphane content being higher in young buds than mature buds, blossom buds, flowers and leaves at bolting stage, and this result was also supported by qRT-PCR. So up-regulation of the *CYP79F1* gene might directly affect glucoraphanin accumulation [22, 31]. In growing and developing buds, because there is no up-regulation of the *CYP79F1* gene, so the intermediates and precursors of glucoraphanin are gradually consumed. Therefore, the results of our study support de novo synthesis of glucoraphanin in young buds. Recent studies have identified two mechanisms of glucosinolate metabolism in plants: transport and de novo synthesis. The first mechanism is the transport of glucosinolate via the phloem from mature leaves to inflorescences and fruits [66, 67]. Other studies have shown that reproductive organs are likely to generate specific and unique glucosinolates by de novo synthesis in these organs [22, 68]. In fact, divergent glucosinolate composition of seeds and other organs have been widely detected, and there are obviously different amounts in the seeds, higher than the other oranges, which also supports the possibility of de novo synthesis in reproductive organs [69]. Our study provided for evidence in synthesis of glucosinolate in reproductive organs.*St5b-2* is numbered K11821 in the KEGG orthology pathway, and it is responsible for tryptophan metabolism, glucosinolate biosynthesis, biosynthesis of secondary metabolites, and 2-Oxocarboxylic acid metabolism. In our study, this gene referred to 4-methylthiobutyl-glucosinolate biosynthesis. According to sequence analysis, another gene in this family, *ST5a-1*, has similar function in tryptophan metabolism, glucosinolate biosynthesis, biosynthesis of secondary metabolites, and 2-Oxocarboxylic acid metabolism. *ST5a-1* and *St5b-2* have been reported in *B*. *rapa*, and their sequences have been analyzed by shotgun sequencing, but still no similar sequence was found in broccoli [35, 37]. In this result, there was a lower level of gene expression in developmental buds compared with in leaves. This might indicate that it supported the accumulation of 4-methylthiobutyl glucosinolate (glucoerucin) for glucoraphanin generated by oxidation by *FMO GS-OX1* gene [58].

The FMO GS-OX family (flavin-monooxygenase) contains five genes of *FMO GS-OX1*~*5*, two genes of *FMO GS-OX2* and *FMO GS-OX5* [70–72], and *FMOGS-OX1* has been identified as an enzyme in the biosynthesis of aliphatic glucosinolates in *Arabidopsis*, catalyzing the S-oxygenation of methylthioalkyl to methylsulfinylalkyl glucosinolate. In sulforaphane synthesis, *FMOGS-OX1* catalyzes the conversion of 4-methylthiobutyl glucosinolate (glucoerucin) to 4-methylsufinylbutyl glucosinolate (glucoraphanin), the precursor of sulforaphane [72–73]. Five FMO genes At1g65860 (*FMO GS-OX1*), At1g62540 (*FMO GS-OX2*), At1g62560 (*FMO GS-OX3*), At1g62570 (*FMO GS-OX4*), and At1g12140 (*FMO GS-OX5*) have been found within a subclade of the FMO phylogeny [31, 57]. In the study, the gene expression of *FMO GS-OX1* was at a low level in developing buds comparing to in leaves, which was similar to the genes of *MAM1* and *St5b-2*, suggesting the similar gene expression patterns of *St5b-2* and *FMO GS-OX1*. Most of studies have reported the hydrolysis products of glucosinolate are controlled by epithiospecifier protein (ESP), myrosinase (MY), and potentially free iron and pH [21, 74]. Previous conclusions have shown that the system of glucosinolate hydrolysis is complex, and some results suggest that the ESP runs functions via interactions with myrosinase [32]. Myrosinase can catalyze the hydrolysis of the thioglucoside linkage and release a glucose and an unstable aglycone. The aglycone moiety subsequently rearranges to form various products depending on the aglycone structure, myrosinase, pH, ferrous ion, zinc and magnesium concentrations [24, 75–77]. Our results showed a high consistency of the gene expression among *MY*, *FMO GS-OX1*, *St5b-2* and *MAM1*. Therefore, the correlations of ten genes in expression level and the contents of sulforaphane and glucoraphanin were analyzed by Pearson correlation test. The result revealed that six genes of *MAM1*, *St5b-2*, *FMO GS-OX1*, *AOP2*, *ESP* and *ESM1* were highly correlated with correlation coefficients from 0.887 to 0.999 (*P*<0.01) (Tables 6 and 7). From the contents and consistent changes of gulcoraphanin and sulforaphane, it could be proved that myrosinase and ESP had not influence on sulforaphane generation at bolting stage.

**Table 6:** The correlation analysis of sulforaphane contents and related genes in different organs.

Pearson Correlation	Sulforaphane	*MAM1*	*IMS2*	*CYP79F1*	*FMO GS-OX1*	*AOP2*	*AOP3*	*MY*	*St5b-2*	*ESP*	*ESM1*
Sulforaphane	1	-0.61	-0.373	0.796	-0.581	-0.51	0.125	-0.468	-0.63	-0.141	0.039
*MAM1*	-0.61	1	0.06	-0.198	.994**	.967**	-0.484	.979**	.999**	0.868	0.757
*IMS2*	-0.373	0.06	1	-0.022	0.093	-0.002	0.828	-0.002	0.055	-0.124	-0.144
*CYP79F1*	0.796	-0.198	-0.022	1	-0.123	-0.045	0.248	-0.015	-0.224	0.282	0.464
*FMOGS-OX1*	-0.581	.994**	0.093	-0.123	1	.985**	-0.443	.990**	.993**	.887*	0.79
*AOP2*	-0.51	.967**	-0.002	-0.045	.985**	1	-0.502	.992**	.967**	.907*	0.828
*AOP3*	0.125	-0.484	0.828	0.248	-0.443	-0.502	1	-0.498	-0.493	-0.494	-0.426
*MY*	-0.468	.979**	-0.002	-0.015	.990**	.992**	-0.498	1	.975**	.939*	0.862
*St5b-2*	-0.63	.999**	0.055	-0.224	.993**	.967**	-0.493	.975**	1	0.855	0.74
*ESP*	-0.141	0.868	-0.124	0.282	.887*	.907*	-0.494	.939*	0.855	1	.980**
*ESM1*	0.039	0.757	-0.144	0.464	0.79	0.828	-0.426	0.862	0.74	.980**	1

Note: *. Correlation is significant at the 0.05 level (2-tailed) and

**. Correlation is significant at the 0.01 level (2-tailed).

**Table 7:** The correlation analysis of glucoraphanin contents and related genes in different organs.

Pearson Correlation	glucoraphanin	*MAM1*	*IMS2*	*CYP79F1*	*FMO GS-OX1*	*AOP2*	*AOP3*	*MY*	*St5b-2*	*ESP*	*ESM1*
glucoraphanin	1	-.634	-.444	.768	-.587	-.473	.061	-.470	-.647	-.183	.000
*MAM1*	-.634	1	.060	-.198	.994**	.967**	-.484	.979**	.999**	.868	.757
*IMS2*	-.444	.060	1	-.022	.093	-.002	.828	-.002	.055	-.124	-.144
*CYP79F1*	.768	-.198	-.022	1	-.123	-.045	.248	-.015	-.224	.282	.464
*FMOGS-OX1*	-.587	.994**	.093	-.123	1	.985**	-.443	.990**	.993**	.887*	.790
*AOP2*	-.473	.967**	-.002	-.045	.985**	1	-.502	.992**	.967**	.907*	.828
*AOP3*	.061	-.484	.828	.248	-.443	-.502	1	-.498	-.493	-.494	-.426
*MY*	-.470	.979**	-.002	-.015	.990**	.992**	-.498	1	.975**	.939*	.862
*St5b-2*	-.647	.999**	.055	-.224	.993**	.967**	-.493	.975**	1	.855	.740
*ESP*	-.183	.868	-.124	.282	.887*	.907*	-.494	.939*	.855	1	.980**
*ESM1*	.000	.757	-.144	.464	.790	.828	-.426	.862	.740	.980**	1

Note: *. Correlation is significant at the 0.05 level (2-tailed) and

**. Correlation is significant at the 0.01 level (2-tailed).

So far, three *AOP2* genes have been identified in *B*. *oleracea*, two are non-functional due to the presence of premature stop codons, and no *AOP3* gene has been found [35]. In contrast, all three *AOP2* copies are functional in *B*. *rapa*, resulting in conversion of glucoraphanin into gluconapin, which explains why glucoraphanin is abundant in *B*. *oleracea*, but not in *B*. *rapa* [31, 35]. *AOP3* also does not exist in *B*. *rapa*, which contains three *AOP* loci orthologs, each containing two tandem duplicated genes [21, 60]. Studies in *Arabidopsis* have shown differential *AOP* leaf expression, whereby a particular accession expresses either *AOP2* or *AOP3* but not both [70, 78], which has been reported to be due a complete inversion of the *AOP2* and *AOP3* structural genes in some accessions, causing the *AOP3* gene to be expressed from the *AOP2* promoter [79]. This conclusion is in conflict with the absence of an *AOP3* gene in cabbage [35], but our results support this conclusion in *Arabidopsis* based on *AOP3* gene expression in this study.

According to the gene expression patterns of *AOP2* and *AOP3*, it was found that *AOP2* gene, likely *MY*, *FMO GS-OX1*, *St5b-2* or *MAM1*, showed a lower level of expression in developing buds than in leaves (Fig 5). However, there was significantly higher *AOP3* expression in developmental buds compared to leaves (Fig 5), the highest being in flowers, followed by young buds, mature buds and buds one day before flowering, and leaves were at the lowest level. *AOP3* should be present in broccoli plant, however it was detected in the expression of the *AOP3* domain, which might provide us new evidence for explaning the diversity of sulforaphane in different broccoli organs. Meanwhile the *AOP3* gene plays a role in hydroxylation of glucoraphanin, which might partly explain why there was lower accumulation of glucoraphanin in flowers compared to the other developmental buds, resulting in low concentration of sulforaphane [41, 60].

### Plant hormones in pathways affecting glucoraphanin accumulation

Some studies have reported glucoraphanin and sulforaphane are influenced by genotype, developmental stages and environment effects, and others state that plant hormones, such as IAA and jasmonic acid (JA), also affect glucoraphanin production, resulting in changes in sulforaphane levels in broccoli [22–24, 57]. Several reports indicate that the loss function of *CYP79F1* in mutations could end the formation of short-chain methionine-derived glucosinolates, but increase the amounts of IAA and cytokinin [80]. Glucosinolate syntheses also conversely affect the levels of auxin and cytokinin [27, 80]. JA is an elicitor and signaling molecule for glucosinolate biosynthesis, it has been shown to enhance both the production of indolic glucosinolates and their biosynthetic gene transcript levels in *Arabidopsis*, and the accumulation of glucoraphanin in broccoli could be up-regulated by JA related genes [38, 81].

In this study, plant hormone signal transduction was analyzed by RNA sequencing, and there were 95, 89, 97 and 86 corresponding DEGs with the same 521 background genes based on the developing buds (LN_B1- 4) versus leaf expression. According to the differences in plant hormone signal transduction gene expression among four organs in developmental buds, 2 DEGs were found in the auxin signaling pathway, one was up-regulated (*Bo5g027930*) only in young buds. This finding reminded us the association of young buds with higher sulforaphane content and higher expression of *Bo5g027930* only occurring in this organ, which might provide evidence for the importance of *CYP79F1*. The specific mechanism driving these observations still needs further research. The other auxin signaling gene was down regulated (*Bo9g151530*), and it occurred in buds one day before flowering and flowers. Thus, it could be inferred that different auxin response might affect the accumulation of glucoraphanin [27, 41].

In the cytokinin signaling pathway, 3 up-regulated genes and 2 down-regulated genes were different in developing buds. A total of 3 up-regulated genes, *Bo8g091410*, *Bo3g107060* and *Bo3g035110*, only showed higher expression in young buds and were absent in the remaining developing buds. In contrast, 2 genes, *Bo5g027070* and *Bo8g059410*, were down-regulated in buds one day before flowering and flowers, and absent from in young and mature buds. These 5 genes belong to the two-component response regulator ARR-A family, which might be potential genes in affecting glucoraphanin generation [29].

## Conclusions

In the study, it was found that *CYP79F1* plays a fundamental and direct role in sulforaphane production of inflorescences at differential developmental stages, and a low expression level resulted in a decrease of this compound or the precursor glucoraphanin due to competition for the intermediates, such as 2-oxo-6-methylthihexanoic acid or 4-methylthiobutyl (glucoerucin). These genes of *MAM1*, *MAM3*, *St5b-2*, *FMO GS-OX1* were in favor of glucoraphanin, *MY*, *ESP* and *ESM1* played a high efficiency function in sulforaphane generation although with low expression level in this stage. At the same time, the plant hormones auxin and and cytokinin might affect glucoraphanin accumulation. The knowledge gained from this study provides a way to study different molecular mechanisms and the diversity of sulforaphane in different organs during broccoli development stages.

## Supporting information

S1 Fig The most enriched GO terms. (TIF)

S2 Fig Patterns of gene expressions in the developmental buds and leaves of B52 by STEM analysis (*P* < 0.05). The green line represents the expression pattern of all the genes. The number of genes belonging to each pattern is labeled above frame.
(TIF)

S3 Fig The distribution of clean reads, containing N, low quality and adapter related reads in the raw reads. (TIF)
