# Supplementary figures and images for: Transcriptome reveals the gene expression patterns of sulforaphane metabolism in broccoli florets
Source: PLoS One. 2019 Mar 25;14(3):e0213902. doi: 10.1371/journal.pone.0213902 (PMC6433254; doi:10.1371/journal.pone.0213902)

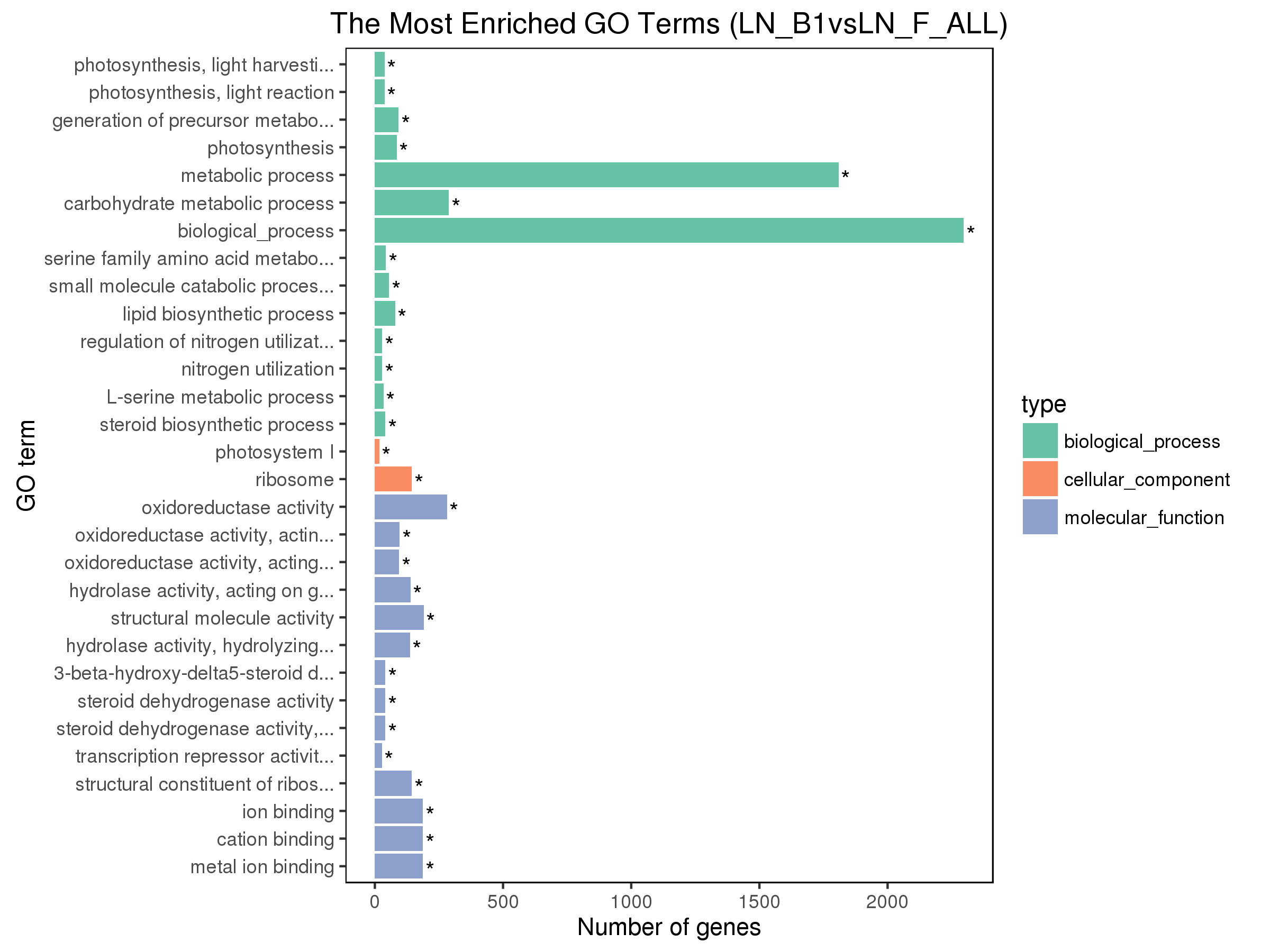

Supplement: S1 Fig — (TIF) [file pone.0213902.s001.tif]

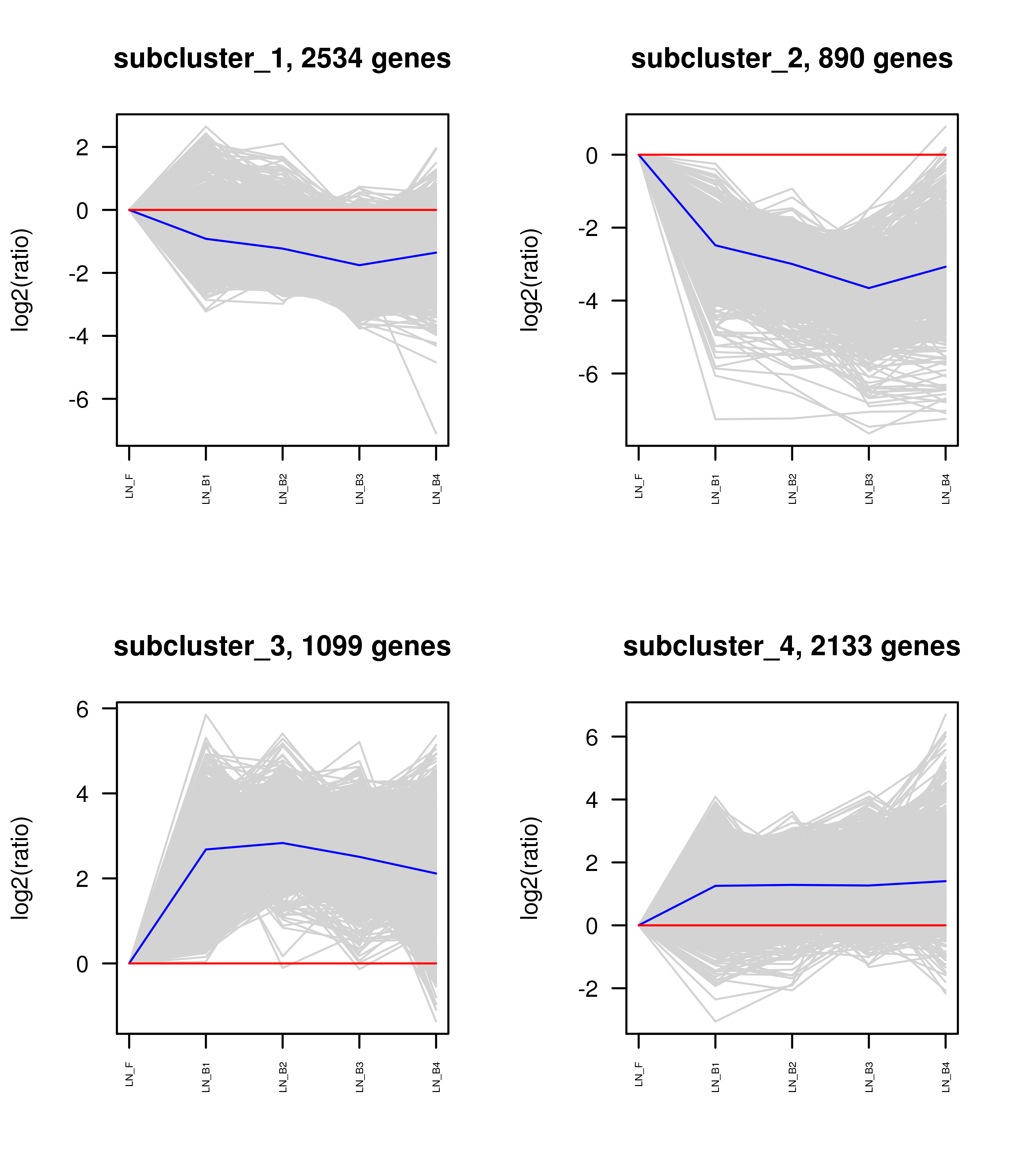

Supplement: S2 Fig — The green line represents the expression pattern of all the genes. The number of genes belonging to each pattern is labeled above frame. (TIF) [file pone.0213902.s002.tif]

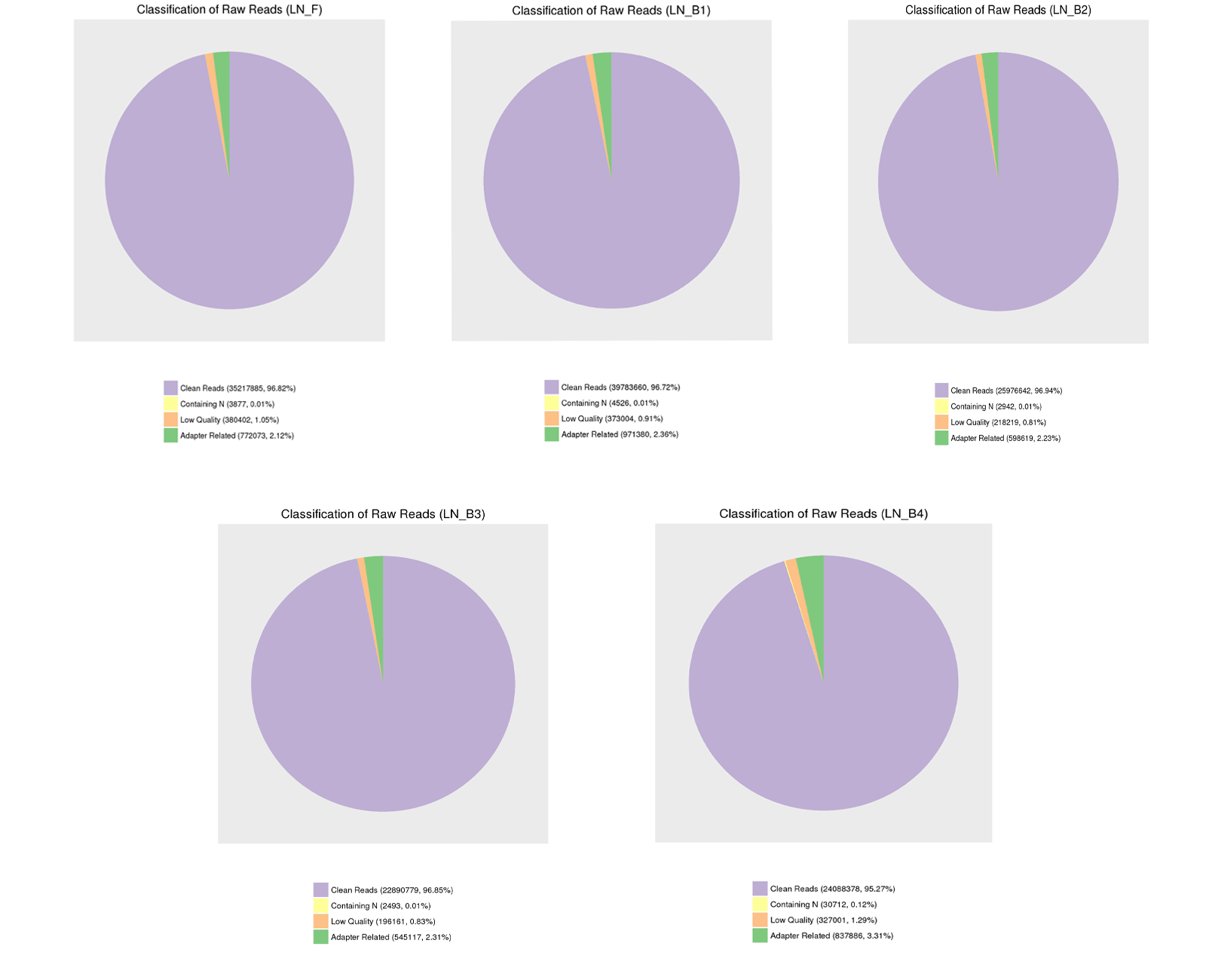

Supplement: S3 Fig — (TIF) [file pone.0213902.s003.tif]
